# Clinical Lectures on Diseases of the Ear

**Published:** 1872-08

**Authors:** E. L. Holmes

**Affiliations:** Professor of Ophthalmology and Otology, Rush Medical College, Surgeon to the Illinois Charitable Eye and Ear Infirmary; Chicago


ARTICLE II.—*Clinical Lectures on Diseases of the Ear.* By E. L. HOLMES, M.D., Professor of Ophthalmology and Otology, Rush Medical College, Surgeon to the Illinois Charitable Eye and Ear Infirmary.

THE MEMBRANA TYMPANI.

*Gentlemen:*

You have all repeatedly observed, as patients have presented themselves here for treatment, the membrana tympani in various conditions of the ear. In the examination of the objective symptoms in any aural disease, the appearance of this membrane will be of more importance, in diagnosis, than that of any other portion of the organ.

First, let us recur to the appearance of the normal membrane, which, as you can see in examining these four healthy individuals of different ages, may vary in different persons, in regard to "its relative position, inclination, form, size, color, curvature, lustre, degree of transparency, and texture." By observing carefully and frequently these peculiarities in health, you can readily detect any abnormal change which may exist.

The membrane consists of three principal layers: the inner, a mucous membrane—the outer, the epidermis—and the middle; which last may be divided again into radiating and circular fibres.

You are especially to observe the condition of the little ridge produced by the handle of the malleus, and the triangular light spot. The former extends from near the middle of the membrane upward and forward to the periphery. The latter extends from near the centre of the membrane downward and forward to the periphery.

Thus you perceive the membrana tympani is divided into two portions: the smaller triangular portion, anterior to the light spot and handle of the malleus; and the larger portion, posterior to these boundaries. By observing these few relations, you have the most important outlines in locating all abnormal changes.

The vessels of the membrane are quite abundantly distributed in its outer and its mucous layers. The branches which run parallel with the handle of the malleus, are larger than the others. Any inflammatory action very near the membrane, is certain to produce congestion of these vessels, which should be an object of especial notice in examinations of the ear.

The nerves of the membrane are confined, principally, to its outer layers. The chorda tympani, a branch of the facial nerve, passes between the malleus and incus, across the anterior portion of the periphery of the membrane, but distributes no branches to it.

The physiology of the membrana tympani is simple. It receives the undulations of the atmosphere and communicates them through the chain of bones to the fluid of the labyrinth. And yet, you should remember, that large portions of the membrane may be destroyed without much impairing hearing, provided the mucous membrane, and especially the stapes and membrane of the foramen rotundum, remain intact.

The functions of the membrana tympani, through the influence of the tensor tympani and the stapedius muscles, seem to be, to a certain degree, analogous to the accommodative apparatus of the eye. These muscles appear to assist in the act of listening, although I believe they are far less active than the uliary muscle in the eye.

In studying the diseases of the membrana tympani, you should bear in mind that an idiopathic inflammation of this structure is almost absolutely unknown. Whenever you perceive any change in its structure, from disease, you may rest almost certain that it is dependent on disease of some other portion of the ear, either the external meatus or middle ear and eustachian tubes.

The most common abnormal appearances which you will meet, are vascularity, unevenness of surface (collapse), the handle of the malleus being remarkably distinct, perforation, and want of transparency.

In regard to perforations, I can only say that they occur chiefly from ulcerative process, dependent on inflammation of the external meatus, or middle ear. The opening is sometimes in such a position that it cannot be seen. One of the most important aids in diagnosticating perforation, is simple auscultation while the patient, closing the nostrils and mouth, forcibly expires, which produces a peculiar whistling sound. Whenever, on looking into the external meatus, you perceive a pulsating movement of the purulent discharge collected on the membrane, you can be almost positive that there is a perforation.

It is comparatively seldom that you will meet with a case of perforation without more or less otorrhœa. The case before you is exceptional: you perceive a well-defined oval perforation, as a clear black spot, just behind the centre of the membrane. There is, apparently, not the slightest moisture around it. As the patient, closing the nostrils and mouth, forcibly expires, you hear distinctly the sound as the air passes through the ear. The perforation is, probably, the result of active inflammation which occurred years ago, but scarcely impairs hearing.

You will, occasionally, meet with ruptures and punctures of the membrana tympani. It is a matter of interest for you to know that such wounds, unless attended with injury of other portions of the ear, almost always heal without treatment. It is well, however, to remove coagula by gently syringing with tepid water. The patient should avoid violent efforts in blowing the nose.

Before attempting to heal perforations produced by inflammation, you should relieve the primary disease, and especially the otorrhœa. But this involves the subject of treatment of the middle ear, which we shall discuss hereafter. After the discharge has ceased, small openings will sometimes heal spontaneously. Large ones are occasionally induced to heal by applications of caustic to the edges of the opening. You must not, however, expect too much, for large openings, and not unfrequently small ones, are very difficult to close.

It remains for me simply to refer you, for future study, to the subject of artificial membrana tympani. The one I present to you is a simple disc of thin rubber, to the centre of which is attached a slender stem; this is inserted to a certain distance in the external meatus. The exact position of this disc, as also that of a tuft of cotton which is often used to improve hearing, is learned by experience by the patient.

